# Tyrosine Hydroxylation in Betalain Pigment Biosynthesis Is Performed by Cytochrome P450 Enzymes in Beets (*Beta vulgaris*)

**DOI:** 10.1371/journal.pone.0149417

**Published:** 2016-02-18

**Authors:** Rasika Sunnadeniya, Alexander Bean, Matthew Brown, Neda Akhavan, Gregory Hatlestad, Antonio Gonzalez, V. Vaughan Symonds, Alan Lloyd

**Affiliations:** 1 Department of Molecular Biosciences, The University of Texas at Austin, Austin, Texas, United States of America; 2 Institute of Fundamental Sciences, Massey University, Palmerston North, New Zealand; University of Illinois at Urbana-Champaign, UNITED STATES

## Abstract

Yellow and red-violet betalain plant pigments are restricted to several families in the order Caryophyllales, where betacyanins play analogous biological roles to anthocyanins. The initial step in betalain biosynthesis is the hydroxylation of tyrosine to form L-DOPA. Using gene expression experiments in beets, yeast, and *Arabidopsis*, along with HPLC/MS analysis, the present study shows that two novel cytochrome P450 (CYP450) enzymes, CYP76AD6 and CYP76AD5, and the previously described CYP76AD1 can perform this initial step. Co-expressing these CYP450s with DOPA 4,5-dioxygenase in yeast, and overexpression of these CYP450s in yellow beets show that CYP76AD1 efficiently uses L-DOPA leading to red betacyanins while CYP76AD6 and CYP76AD5 lack this activity. Furthermore, CYP76AD1 can complement yellow beetroots to red while CYP76AD6 and CYP76AD5 cannot. Therefore CYP76AD1 uniquely performs the beet R locus function and beets appear to be genetically redundant for tyrosine hydroxylation. These new functional data and ancestral character state reconstructions indicate that tyrosine hydroxylation alone was the most likely ancestral function of the CYP76AD alpha and beta groups and the ability to convert L-DOPA to cyclo-DOPA evolved later in the alpha group.

## Introduction

Betalains are water-soluble nitrogenous pigments classified as the red-violet betacyanins and yellow betaxanthins. These pigments variously accumulate in flowers, fruits, and vegetative tissues of plants in most core families of the Caryophyllales order, and where they exist, betalains exclusively replace anthocyanins. The betacyanins play important and analogous roles to anthocyanins in attracting pollinators and seed dispersers. While the structural and regulatory genes of anthocyanin biosynthesis are well known, betalain biosynthesis has been poorly understood at the molecular level creating a phylogenetic mystery surrounding their origin(s).

**Fig 1:**
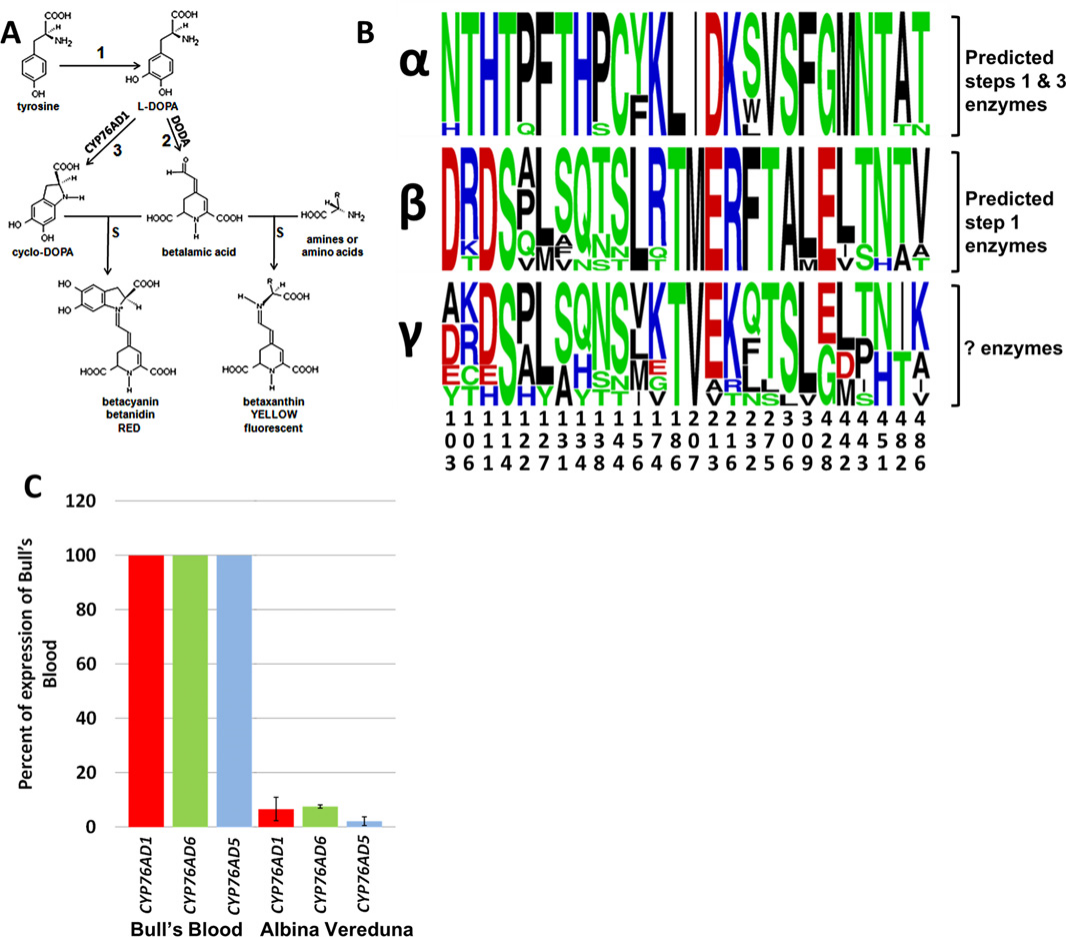
Identification of candidate genes for tyrosine hydroxylation. A. Biosynthetic scheme for betacyanin and betaxanthin production. Tyrosine hydroxylation is step 1. B. LOGO analysis of the three CYP76AD subfamily clades. C. qRT-PCR analysis of the beet CYP76AD1, CYP76AD5, and CYP76AD6 genes in non-transgenic beets. Bull’s Blood is a very dark red table beet and Albina Vereduna is a white table beet. Bull’s Blood is YY, expressing the dominant, high-pigment Y allele of BvMYB1 while Albina Vereduna is yy.

Much of the biosynthetic pathway involved in betalain biosynthesis has recently been described (Fig 1A), yet the enzyme(s) that perform the initial step of the pathway, the hydroxylation of L-tyrosine to L-3,4-dihydroxyphenylalanine (L-DOPA), have largely remained unknown. Once L-DOPA is formed it can either be converted to betalamic acid (BA; step 2) by DOPA 4,5-dioxygenase (DODA) [1–4] or to cyclo-DOPA (step 3) [4,5]. BA is the key intermediate forming the common chromophore of all betalains. BA can spontaneously condense with amino acids or other amine groups to form yellow fluorescent betaxanthin pigments [6] or spontaneously condense with cyclo-DOPA to form red betacyanin pigments [7]. Earlier work identified the single enzyme responsible for converting L-DOPA to cyclo-DOPA in *Beta vulgaris* (beet) as BvCYP76AD1 [4], which is absolutely necessary to make red betalain pigments in this species.

Investigators have proposed the hydroxylation of L-tyrosine to L-DOPA was performed by the monophenolase activity of a tyrosinase (or a polyphenoloxidase-PPO) [7,8]. There are reports of PPO transcripts that increase with betalain pigment accumulation in pokeweed fruits (*Phytolacca americana*; Phytolaccaceae [9], and PPOs have been purified from beet [10] and red Swiss Chard (same species as beet) [11]. A tyrosinase with both monophenolase and diphenolase [7] activities, and one with just monophenolase activity [12] have been purified from *Portulaca grandiflora* (Portulacaceae), forming L-DOPA from tyrosine and cyclo-DOPA from L-DOPA *in vitro*. Mushroom tyrosinases have been shown to perform the tyrosine hydroxylation *in vitro* and in transgenic plant tissues [13]. Even though these findings suggest PPO involvement in betalain biosynthesis, others point out that there is no direct evidence for PPO involvement in betalain biosynthesis *in vivo* [13–15]. Thus, until recently there was no direct evidence for identification of the genes and enzyme(s) responsible for performing the key first step, tyrosine hydroxylation, in the betalain biosynthesis pathway, representing the only ring biosynthetic step not molecularly identified. DeLoache et al. [5] recently described a yeast synthetic biology assay in which CYP76AD1 was shown to have both tyrosine hydroxylating activity and the ability to produce cyclo-DOPA from L-DOPA [4]. Polturak et al. [16] subsequently showed that CYP76AD6 is also capable of tyrosine hydroxylation.

Here, the search for the genes/enzymes responsible for tyrosine hydroxylation in the beet betalain biosynthetic pathway is described. Two novel beet cytochrome CYP450 genes (*CYP76AD5* and *CYP76AD6*) were identified with mRNA levels positively correlated with high betalain levels. These two genes are in the beta clade of CYP76AD genes described by Brockington et al. [17], while *CYP76AD1* is in the alpha clade. The newly identified CYP76AD5 and CYP76AD6 and the previously characterized CYP76AD1 [4,5] are all able to perform tyrosine hydroxylation leading to betalain biosynthesis not only in beet tissues, but also in yeast and Arabidopsis. However, while CYP76AD1 is also able to produce cyclo-DOPA, CYP76AD5 and CYP76AD6 do not have this activity. These differential clade biochemical activities are verified by showing that alpha and beta clade members, CYP76AD3 and CYP76AD15 respectively, from a representative of a different family, *Mirabilis jalapa* (Nyctaginaceae), have the same activities as alpha and beta clade members from beets (Amaranthaceae). The maintenance of both alpha and beta CYP76AD proteins would allow plants to differentially regulate these genes to produce different ratios of yellow and red pigment, thus allowing yellow, orange, red, or patterned pigmentation.

## Materials and Methods

### Cytochrome P450 and PgDODA Cloning and Sequence

The *CYP76AD1* (Genbank HQ656023; see S1 Table for list of genes) sequence was obtained from Roche/454 pyrosequencing performed on cDNA from 7-day-old *Beta vulgaris* cv. W357B seedling hypocotyls [4]. cDNA from 7-day-old hypocotyls of three white beet cultivars, table beet varieties Blankoma and Albina Vereduna, and sugar beet variety C869, was sequenced by SOLiD (Applied Biosystems) nextgen sequencing. The SOLiD RNAseq reads were mapped separately on to the assembled contigs of the red table beet cultivar W357B Roche/454 reference sequence database [4]. The approximate fold changes of gene expression from red to each white beet variety was calculated as follows. The ratio of the number of reads for a particular contig over the total number of RNAseq reads in that beet variety was determined. Then the fold difference in these ratios was determined in red vs. white.

In order to search for candidate genes for the betalain pathway tyrosine hydroxylation step, specifically CYP450s or PPOs, genes with relatively high expression levels in red beets compared to white beets were identified. Five-fold change in expression level was used as the base line to identify candidate genes. Expression levels of candidates were then tested through qPCR.

Previous work reported the identification and cloning of *CYP76AD1*, which lies at the R locus [4,18]. CYP76AD5 and CYP76AD6 were both found to be differentially expressed in white and red beet RNAseq databases. The cDNA sequences were amplified by PCR from cDNA made from red hypocotyls of 7-day-old W357B seedlings. Primer sequences: CYP76AD5StartF 5’ATGGATAACACTACACTTGCATTGATACTTTCTTC3’; CYP76AD5StopR 5’TTATTTCCTAGGTACGGGAATAACTTGGAGAGGC3’; CYP76AD6StartF 5’ATGGATAACGCAACACTTG3’; CYP76AD6StopR 5’CCAGTTCCCAGAAACTAG3’. Gateway recombination sequences were included in all primers but are not shown. The products were cloned into pDONR222 (Invitrogen) and sequenced: *CYP76AD5* (1506 bp; Genbank KM592961), *CYP76AD6* (1500 bp; Genbank KM592962). These clones were recombined into the plant TDNA vector pB7WG2 [19]. This creates p35S::gene for overexpression of the *CYP76AD5*, and *CYP76AD6* coding regions in plants. These plasmids were transformed into *Agrobacterium rhizogenes* ARqua1 [20] for beet transformation, which produces transformed hairy root tissues, or *Agrobacterium tumefaciens* GV3101 [21] for Arabidopsis transformation. A p35S::GUS construct was used as a control.

Cloning of the *Portulaca grandiflora* DODA cDNA (*PgDODA*) into pDONRZeo and subsequently into the yeast vectors, pVV214 or pVV200 [22] has been described [4]. To express PgDODA in plants, the cDNA was recombined into the plant TDNA vector pK7WG2 [19] to make p35S::PgDODA. This was transformed into *A*. *tumefaciens* GV3101 [21] for Arabidopsis transformation.

The cDNA sequences for *CYP76AD3* and *CYP76AD15* were amplified from 7-day-old red 4 o’clock seedling cDNA using the following primers: CYP76AD3StartF 5’ATGGATTTCTTAACCCTTGTCATG3’; CYP76AD3StopR 5’TCAATATTTAATAAGAGGAATAATCTC3’; CYP76AD15StartF 5’ACGTTCAAGCATAGACTAACC3’; CYP76AD15StopR 5’TGATTATCTCCCAACCATCG3’. Gateway recombination sequences were included in all primers but are not shown. The products were recombined into pDONR222 (Invitrogen) and sequenced: *CYP76AD3* (1680 bp; Genbank HQ656026) *CYP76AD15* (1515 bp; Genbank KM516798). These were recombined into pVV214 for yeast expression.

### Over Expression in Beet Hairy Root Cultures

Seeds of yellow beet cultivar Golden Globe (from W. Atlee Burpee & Co.) were sterilized and germinated on MS media with 3% sucrose in petri plates. Overnight liquid LB cultures of the *A*. *rhizogenes* strains were placed into a 1 ml tuberculin syringe. A 30-gauge needle was used to make wounds on the hypocotyls of 10-day-old seedlings, injecting the *A*. *rhizogenes* culture into the wounds. Inoculated seedlings were maintained on MS media with 3% sucrose for 2 days and then transferred to the same media with Timentin 200mg/L to kill the bacteria. Plates were incubated at 22°C in continuous light. “Hairy roots” emerged from wound sites on the hypocotyl in about 2–3 weeks. Roots were excised from the wound site and maintained on MS with 3% sucrose and 200 mg/L Timentin. At least ten independent transgenic lines with consistent phenotype were produced for each construct.

### Arabidopsis Transformation

*Arabidopsis thaliana* mutant *ttg1-1* in the L*er* ecotype (from the Arabidopsis Biological Resource Center) was used for all experiments because it does not produce anthocyanin pigments. Arabidopsis plants were transformed by vacuum infiltration with the described constructs in *A*. *tumefaciens* GV3101 [21].

### Quantitative RT-PCR

For gene expression analysis, tissue was collected from 7-day-old untransformed hypocotyls of the beet cultivars Bull’s Blood (red; from Johnny’s Selected Seeds) and Albina Vereduna (white; from John Scheepers Kitchen Garden Seeds) or of transformed hairy roots generated on Golden Globe by overexpressing *CYP76AD1*, *CYP76AD5*, or *CYP76AD6*. Total RNA was extracted using Qiagen plant RNeasy mini kit and used in 20 μL reverse transcriptase reactions using 1 μg RNA and 1 μg oligo-dT. qRT-PCR was performed as described [23]. Four technical replicates were performed for each target from each biological replicate and for actin controls using 400 nM of the appropriate qRT-PCR primers: BvACT (Genbank HQ656028) 5'TCTATCCTTGCATCTCTCAG3' and 5'ATCATACTCGCCCTTGGAGA3'; CYP76AD1RTF 5' CTTTTCAGTGGAATTAGCCCACC3' and CYP76AD1RTR 5'CCCAATATCTTCCATAATGTTCCA3'; CYP76AD5RTF 5'TCATTTTCATAAGTTCTTCG3' and CYP76AD5RTR 5'CAGTCAACTCATCACTCATC3'; CYP76AD6RTF 5'GCTAACCGAACCATTCCTGA3' and CYP76AD6RTR 5'TTGGACAGCGGAGATTTTTC3'; BvPPO (Genbank KR337592) BvPPORTF 5'AAAACAGTTGAGGCTCCGACC3’ and BvPPORTR 5’GGAAGAGCTTTCATTAAGGC3’. Results were analyzed by the comparative cycle threshold method according to the user manual for the ABI PRISM Sequence Detection System. Each experiment was performed with three biological replicates and mean values of all technical replicates are shown.

### Expression in Yeast and Feeding Assay

The cDNAs of *CYP76AD1*, *CYP76AD6*, *CYP76AD5*, *CYP76AD3*, and *CYP76AD15* in yeast expression vector pVV200 [22], and pVV200 empty vector control were transformed into the yeast strain WAT11 [24]. The WAT11 yeast strain contains a galactose-inducible nicotinamide adenine dinucleotide phosphate (NADPH)–cytochrome P450 reductase from Arabidopsis. Yeast cultures (pVV200/*CYP76AD1*; pVV200/*CYP76AD5*; pVV200*CYP76AD6*; and pVV200/empty) were grown overnight in minimal medium with galactose and supplemented with 100 mg/L leucine, 20 mg/L histidine, and 40 mg/L adenine. The cultures were pelleted by centrifugation. The yeast were resuspended to an OD_600_ of 1.1 in 3 mL of above media with 10 mM tyrosine and 25 mM ascorbic acid.

The WAT11 yeast strain was also co-transformed with the above vector constructs and *PgDODA* cloned into pVV214 [4,22] to make yeast carrying: pVV200/*CYP76AD1* + pVV214/*PgDODA*; pVV200/*CYP76AD6* + pVV214/*PgDODA*; pVV200/*CYP76AD5* + pVV214/*PgDODA*; pVV200/*CYP76AD3* + pVV214/*PgDODA*; pVV200/*CYP76AD15* + pVV214/*PgDODA*; and pVV200/empty vector + pVV214/*PgDODA*. These yeast co-expression cultures were also grown overnight in minimal medium (with galactose and supplemented with 100 mg/L leucine, 20 mg/L histidine and 40 mg/L adenine) and resuspended in 3 mL as above with three separate substrate feedings: 10 mM L-DOPA + 25 mM ascorbic acid; 10 mM tyrosine + 25 mM ascorbic acid; and no enzyme substrate + 25 mM ascorbic acid, for each independent strain. Cultures were grown for 24 hrs after feeding and pelleted by centrifugation and 500 μL of the supernatant was retained for pigment analysis as described below in the pigment chemical analysis section.

### Pigment Chemical Analysis

The preparation of pigment standards for High Performance Liquid Chromatography / Mass Spectrometry (HPLC/MS) analysis was performed by preparative Thin Layer Chromatography (TLC) from red and yellow beet extracts as described [25].

The supernatants from the yeast feeding assays, or plant pigment extracts from transgenic hairy roots overexpressing *CYP76AD1*, *CYP76AD5*, or *CYP76AD6* in beet cultivar Golden Globe, were filtered through a 0.2 μm membrane filter. The filter residue was rinsed with 100% methanol to elute the pigment completely. Eluted pigment was concentrated by vacuum at 30°C and resuspended in 100 μL of 0.1% ascorbic acid. Resuspended pigments were analyzed by HPLC/MS.

Five microliter aliquots of extracts were injected into an Agilent 1260 Infinity HPLC system interfaced with an Agilent 6530 Accurate-Mass QTOF mass spectrometer. The samples were separated on a ZORBAX Eclipse XDB-C18 column (5 μm, 50 x 4.6 mm) using a two-stage LC program. The first stage of the program was a 2 minute isocratic run with a mobile phase of 1% formic acid in water at a flow rate of 0.1 mL/min. The second stage of the program, lasting 6 minutes, included a flow rate gradient from 0.1 mL/min to 0.5 mL/min and a mobile phase gradient of 0–33% B, where mobile phase A is 1% formic acid in water and mobile phase B is 0.1% formic acid in acetonitrile. Mass spec detection of the eluent was performed using positive mode electrospray ionization. Gas temp was 350^0^ C, drying gas flow was 12 L/min, nebulizer pressure was 50 psig, VCap was 5000 V, fragmentor voltage was 200 V, the skimmer was set to 65 V. The mass spec data was acquired scanning the m/z range from 100 m/z to 3200 m/z.

**Fig 2:**
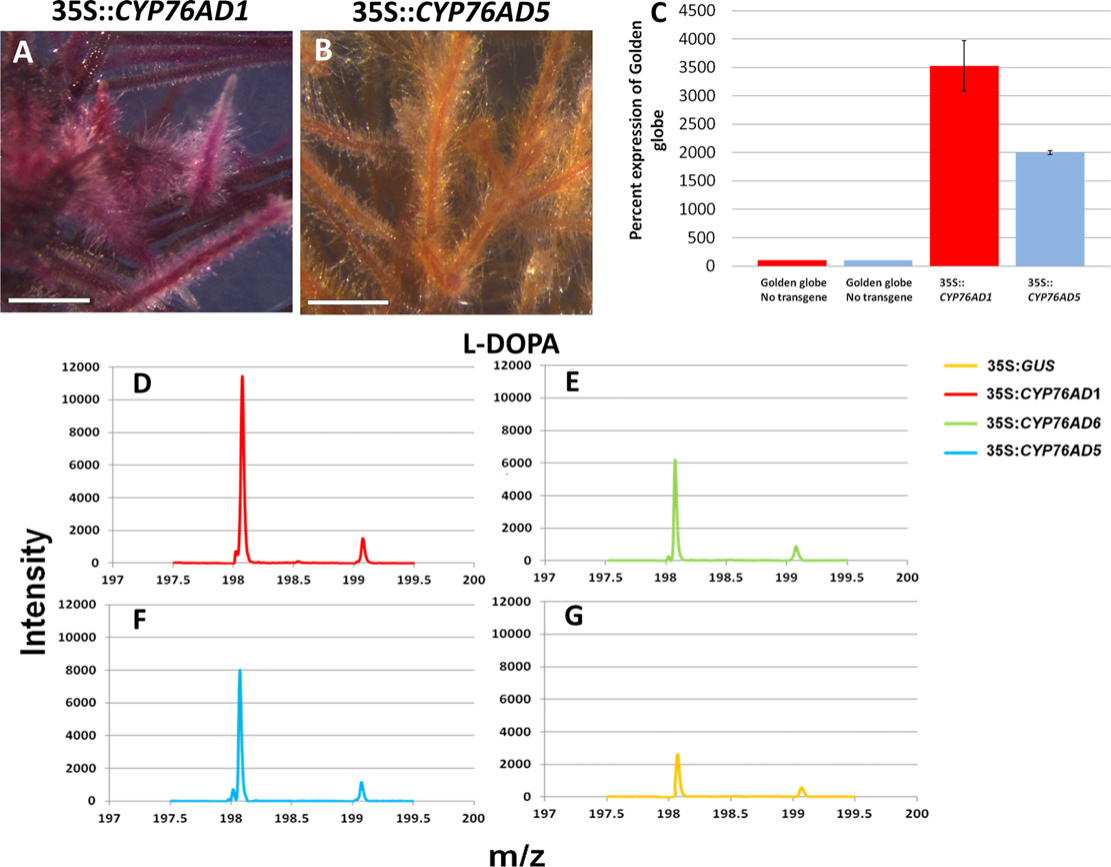
Overexpression of CYP76AD1, CYP76AD6, or CYP76AD5 results in increased L-DOPA content in beet. A. and B. CYP76AD1 complemented, but CYP76AD5 did not complement the *rr* (*CYP76AD1*) beet mutant (Golden Globe) from yellow to red. C. qRT-PCR verified that *CYP76AD1* and *CYP76AD5* were overexpressed in transgenic hairy root cultures. D, E, F, and G. Mass spectrometry analysis of L-DOPA in Golden Globe beet roots overexpressing CYP76AD1, CYP76AD6, CYP76AD5, and GUS, respectively. Error bars are s.d. Each experiment was replicated three times. Scale bars are 3.5 mm.

**Fig 3:**
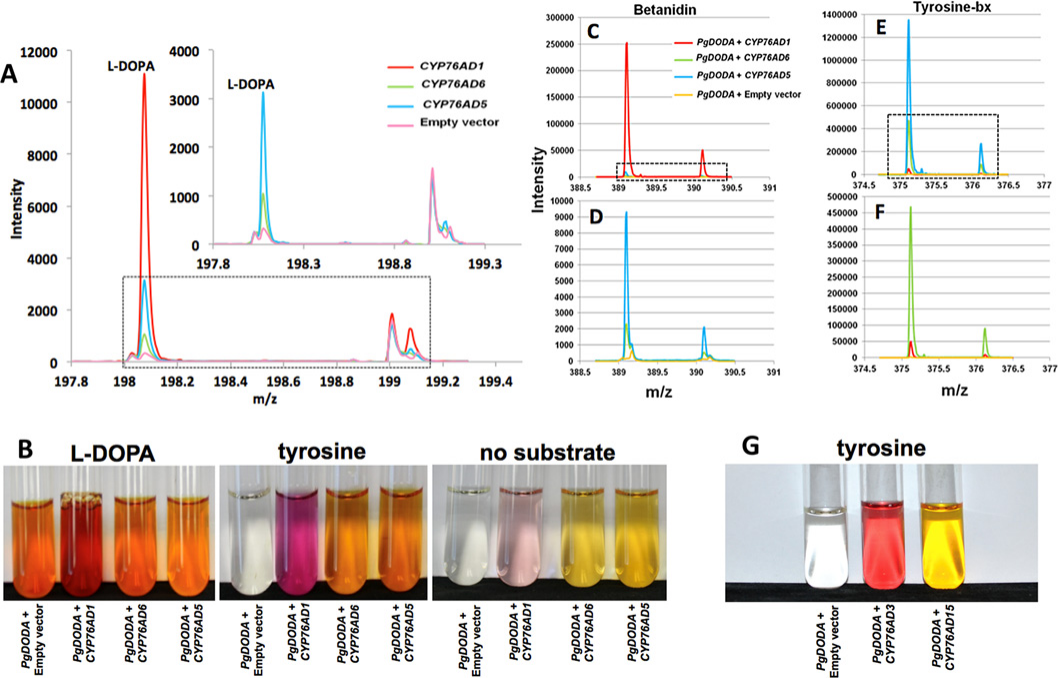
All tested CYP76AD proteins perform tyrosine hydroxylation in yeast. A. HPLC/ms analysis of yeast expressing CYP76AD1, CYP76AD6, and CYP76AD5 individually shows that all will hydroxylate tyrosine to L-DOPA. Inset shows expanded view of lower peaks. Yeast containing empty vector either have weak tyrosine hydroxylation activity or produce a compound that co-migrates with L-DOPA. B to F. Same yeast strains as in A with the addition of PgDODA. B. images of the cultures fed L-DOPA, tyrosine, or no added substrate with the tyrosine-fed panel corresponding to C through F. C and D. HPLC/MS analysis of the red betanidin pigment with the lower peaks for CYP76AD6, CYP76AD5, and empty vector expanded in D. E. and F. HPLC/MS analysis of the yellow tyrosine-betaxanthin pigment with the lower peaks for CYP76AD6, CYP76AD1, and empty vector expanded in F. Note that CYP76AD1 produces large amounts of betanidin and small amounts of betaxanthin. G. Yeast cultures expressing PgDODA alone and PgDODA with CYP76AD3 or CYP76AD15 from *Mirabilis jalapa* and fed tyrosine. CYP76AD3 has CYP76AD1-like activity producing red pigment and CYP76AD15 has CYP76AD6/5-like activity producing yellow pigment.

The exact mass of L-DOPA is 197.188 g/mol. The observed mass for L-DOPA in Fig 2 is 198.076 for CYP76AD1, CYP76AD5, and CYP76AD6, and 198.083 for the 35S:GUS vector. The observed mass for L-DOPA in Fig 3 is 198.076 for CYP76AD1, CYP76AD5, and CYP76AD6, and 198.075 for the empty vector.

### LOGO Analysis of Conserved Amino Acid Sequences Indicative of the CYP76AD Clades

The sequences used to create the phylogeny (S1 Fig; S1 Table) were aligned using Clustal Omega (http://www.ebi.ac.uk/Tools/msa/clustalo/). The alignment was analyzed for residues that appeared to diverge according to the phylogenetic clades (highlighted in S2 Fig). These residues were assembled into a shortened sequence alignment and made into a frequency plot via WebLogo (Fig 1B; http://weblogo.berkeley.edu/logo.cgi). The number for each residue corresponds to its position in CYP76AD1 and the height of the residue is proportional to its appearance within the clade members.

### Phylogenetic Analysis of the Betalain Pathway CYP450s

The cDNA sequences of *BvCYP76AD1*, *BvCYP76AD5*, and *BvCYP76AD6* were each submitted to BLAST searches at NCBI and OneKP to identify orthologs from across the Caryophyllales and beyond. The resulting set of sequences and selected outgroup sequences (members of two other CYP76 subfamilies) were aligned using the MUSCLE alignment tool in GENEIOUS [26] and edited to match reading frames manually. The resulting alignment was assessed in JMODELTEST 2 [27] to determine the best fitting model of molecular evolution. Phylogeny estimation was performed using Bayesian inference as implemented in MRBAYES 3.2 [28] under GTR+I+G. Following 20 million generations, convergence was complete (standard deviation of split frequencies was <0.001) and a consensus tree generated from the trees sampled every 1000 iterations after discarding the first 25% of samples as burn-in (S1 Fig).

Ancestral amino acid sequences were generated for all internal nodes of the CYP76 gene tree using CODEML as implemented in PAMLX version 1.3.1 [29]. Input for the reconstructions included the same gene tree shown in S1 Fig and an amino acid alignment of the same sequences, produced using the MUSCLE alignment option in GENEIOUS version R9 [26]. New ancestral sequences were aligned with the original sequences analyzed in the gene tree and amino acid frequency logo and compared at sites of interest.

## Results

To identify betalain pathway gene/s responsible for tyrosine hydroxylation, RNAseq was performed on three white beet cultivars—Blankoma, Albina Vereduna, and sugar beet cv. C869. Red beets contain a dominant Y betalain regulatory locus allele [18,30] while the white beets are homozygous yy. White beets make betalains but accumulate very low levels of betalain pigmentation compared to red beets.

The reads from the RNAseq data of the white beet varieties were mapped onto a red beet reference sequence database [4] and genes were identified that were both expressed in lesser amounts in white beets as compared to red and might encode tyrosine hydroxylating enzymes, such as CYP450s or PPOs. This candidate gene search revealed two novel CYP450 genes from the same gene family as *CYP76AD1*: *BvCYP76AD5* and *BvCYP76AD6* (Fig 1B, S1 Fig). In the RNAseq database analysis, the expression of CYP76AD5 is down 5, 41 and 9 fold in white beet cultivars Albina Vereduna, Blankoma, and C869, respectively, compared to red beet, while the expression of *CYP76AD6* is down 14, 115 and 6 fold. Meanwhile, known betalain biosynthetic genes, *Beta vulgaris DODA1* (*BvDODA1*) and *CYP76AD1*, are down-regulated many fold in all three white beet varieties: *BvDODA1* is down 71, 207, 57 fold while *CYP76AD1* is down 19, 22, 26 fold in Albina Vereduna, Blankoma, and C869, respectively.

Genes considered to be likely candidates via RNAseq analysis were also subjected to quantitative RT-PCR (qRT-PCR) to confirm the expression differences observed in the RNAseq data. qRT-PCR of *CYP76AD1*, *CYP76AD5* and *CYP76AD6* showed that these genes were expressed at 7%, 2%, and 8% respectively, in Albina Vereduna white beets vs. red beet cultivar Bull’s Blood (Fig 1C).

A single *PPO* (*BvPPO*; genbank KR337592) was also identified in the RNAseq database and in the sugar beet genome [31] and its expression was analyzed in white beets vs. red via qRT-PCR. The expression level of *BvPPO* was 2.1 fold higher in Albina Vereduna and 3.8 fold higher in sugar beet C869 than in the dark red cultivar Bull’s Blood. This lack of correlation between *BvPPO* expression and betalain production does not support a role for this gene in betalain biosynthesis.

cDNAs for *CYP76AD5* and *CYP76AD6* were amplified from red beet seedlings. *CYP76AD5* and *CYP76AD6* have open reading frames of 1506 bp and 1500 bp respectively; the nucleotide identity is 81% while the amino acid identity is 85%. The amino acid identity between *CYP76AD1* and *CYP76AD6* is 72% while the identity between *CYP76AD1* and *CYP76AD5* is 69%.

Previous work showed that CYP76AD1 performs step 3 (Fig 1A) of the betalain pathway converting L-DOPA to cyclo-DOPA [4]. Golden Globe is the yellow beet commonly sold in grocery stores and it carries a mutant allele of *CYP76AD1*, which lies at the R locus. Ectopic expression of CYP76AD1 in Golden Globe complements it from yellow to red. To test whether *CYP76AD5* or *CYP76AD6* can also perform step 3, these genes were overexpressed in Golden Globe tissue via *Agrobacterium rhizogenes* and pigment phenotypes were observed in transformed hairy roots. Overexpression of *CYP76AD1* in yellow beetroots complemented them to red (Fig 2A). Overexpression of *CYP76AD5* (Fig 2B) and *CYP76AD6* (not shown) in yellow beets resulted in yellow, non-complemented roots.

qRT-PCR was performed to verify that the respective genes were overexpressed in all hairy root cultures (Fig 2C). Increases in L-DOPA levels in tissues carrying these transgenes were observed using HPLC/MS analysis (Fig 2D to 2G). The L-DOPA mass spec peak intensity levels (m/z 198) were highest for CYP76AD1, followed by CYP76AD5, and CYP76AD6, in tissues overexpressing these genes respectively, vs. 35S::GUS overexpression control lines, indicating that all three of these genes are able to convert tyrosine to L-DOPA in beets.

*CYP76AD1*, *CYP76AD6*, and *CYP76AD5* were also expressed in yeast to test for tyrosine hydroxylation activity by detecting L-DOPA formation after growing in media containing 10 mM tyrosine (Fig 3A). L-DOPA, the product of tyrosine hydroxylation, was detected with all three CYP450s through HPLC/MS analyses of extracts (Fig 3A). The L-DOPA peak intensity was highest with *CYP76AD1*, followed by *CYP76AD6* and *CYP76AD5* respectively. There is also a very low intensity mass spectrometry peak that co-migrates with L-DOPA in the empty vector control. It is not known if this is L-DOPA or another compound.

The CYP450s were also co-expressed in yeast with the DODA gene (step 2) from *Portulaca grandiflora*, *PgDODA*, to analyze red and yellow betalain pigment formation (Fig 3B to 3F). These were grown in media for 24 hours with: 10 mM tyrosine, the substrate for step 1; or 10 mM L-DOPA, the substrate for both steps 2 and 3 (Fig 1A); or no supplemented feeding. Of the cultures fed L-DOPA: *PgDODA* alone (*PgDODA* + empty vector); *PgDODA* + *CYP76AD6*; *PgDODA* + *CYP76AD5* produced visible yellow to orange pigmentation; while *PgDODA* + *CYP76AD1* produced red pigmentation. Of the cultures fed tyrosine, *PgDODA* + empty vector produced no color; *PgDODA* + *CYP76AD6*; *PgDODA* + *CYP76AD5* produced yellow to orange pigmentation; while *PgDODA* + *CYP76AD1* produced red pigmentation. In the absence of added substrate feeding all four cultures produced colors similar to tyrosine feeding but with lower intensity. Pigments produced without supplemental feeding were undoubtedly through the conversion of native yeast-produced tyrosine to betalain pigments.

The pigments extracted from the four yeast cultures co-expressing DODA and fed tyrosine were analyzed by HPLC/MS to detect the presence of betanidin, the undecorated red betalain pigment, and tyrosine-betaxanthin (tyrosine-bx) as a representative of yellow betaxanthins (Fig 3C to 3F). Betanidin (m/z 389) was detected at very high mass spec intensity in *PgDODA* + *CYP76AD1* as previously reported [4] and interestingly it was also present at very low intensities in *PgDODA* + *CYP76AD6* and *PgDODA* + *CYP76AD5* at about 27 and 100 fold less intensity, respectively (Fig 3C with lower peaks expanded in Fig 3D). These data suggest that CYP76AD6 and CYP76AD5 both have very weak ability to produce cyclo-DOPA, step 3, which is required to produce the betanidin pigment (Fig 3D). Betanidin was absent in the *PgDODA* + empty vector sample, but note that this sample has a small peak that runs at a slightly higher m/z ratio than betanidin. Tyrosine-bx (m/z 375) was detected at high intensities in both *PgDODA* + *CYP76AD6* and *PgDODA* + *CYP76AD5* samples. The tyrosine-bx level was very low in *PgDODA* + *CYP76AD1* and absent in *PgDODA* + empty vector (Fig 3E with lower peaks expanded in Fig 3F).

While expression in yeast clearly indicates the activities of the divergent beet CYP76ADs, these activities were verified by expression in *Arabidopsis thaliana*. Expression of *35S*::*PgDODA* alone results in yellow betaxanthin production only when the plants are fed L-DOPA (data not shown), the same as seen in yeast, and the same that has been previously demonstrated in Arabidopsis [32].

**Fig 4:**
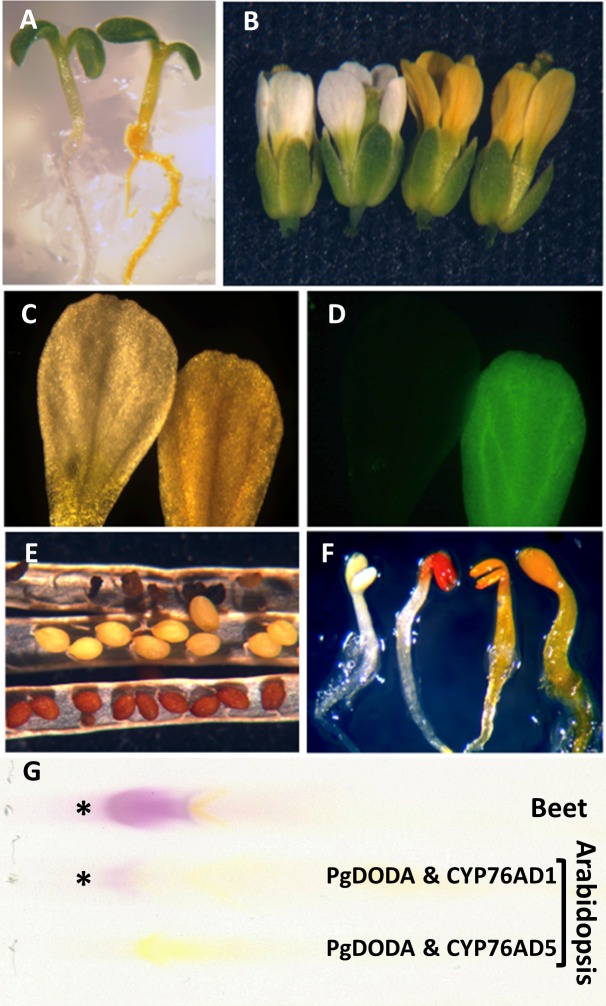
Arabidopsis expressing betalain biosynthetic genes produce visible pigment phenotypes. All Arabidopsis are *ttg1-1* mutant except the wild type L*er* ecotype in panel E. A. left, *35S*:*CYP76AD5* alone, right *35S*:*CYP76AD5* and *35S*:*PgDODA*. B. left two flowers and right two flowers the same as in A. C and D are images of the same two petals. Left and right, the same as in A and B. C. white light, D. excitation with UV irradiation showing fluorescence of betaxanthins. E. mature siliques with seeds; top-*35S*:*CYP76AD5* in *ttg1-1* alone, middle-*35S*:*PgDODA* in *ttg1-1* alone, bottom-wild type L*er*, no transgene. F. seedlings, left to right- *35S*:*CYP76AD1* and *35S*:*PgDODA* unfed; *35S*:*CYP76AD1* and *35S*:*PgDODA* fed 1.5 mM tyrosine; *35S*:*CYP76AD5* and *35S*:*PgDODA* fed 1.5 mM tyrosine; *35S*:*CYP76AD5* and *35S*:*PgDODA* unfed. G. Thin Layer Chromatography of betalain pigments: top-untransformed red beet; middle-Arabidopsis transformed with *35S*:*CYP76AD1* and *35S*:*PgDODA* fed 1.5 mM tyrosine; bottom-Arabidopsis transformed with *35S*:*CYP76AD5* and *35S*:*PgDODA* fed 1.5 mM tyrosine. Asterisks mark the presence of red betacyanins.

Similarly, *35S*::*CYP76AD5* expression alone in Arabidopsis results in no pigmentation in most of the tissues of the living plant (Fig 4A, 4B and 4C left; exception discussed below). When *PgDODA* and *CYP76AD5* are co-expressed in Arabidopsis, the plants develop intense yellow betaxanthin pigmentation (Fig 4A, 4B and 4C right) without any substrate feeding, again repeating the yeast result. This yellow pigment is easily visible in all non-photosynthetic tissues, where chlorophyll does not mask the yellow color. This betaxanthin pigment is also highly fluorescent [33] and can be seen via fluorescence microscopy (Fig 4C and 4D). In addition, it is noted that expression of CYP76AD5 alone causes the seeds to develop black pigmentation and become small and shriveled (Fig 4E), and upon drying at maturity, many of the plants develop this same gray or blackish pigment, mostly on siliques and stems (not shown). This is probably a form of melanin pigment produced from L-DOPA, but the pigment has not yet been analyzed.

Expression of 35S::CYP76AD1 alone does not produce pigmentation in any plant tissues, not even in the seeds (not shown). When PgDODA and CYP76AD1 are co-expressed in *A*. *thaliana*, no pigmentation is visible without feeding, despite the predicted presence of red and/or yellow coloration. However, when 3-day-old seedlings were transferred from germination media to the same media with 1.5 mM tyrosine prior to greening, red pigmentation was observed in the cotyledons and upper hypocotyl (Fig 4F second from left). If they are transferred after the cotyledons have expanded and turned green, the red pigment is not produced. Thin Layer Chromatography (TLC) was used to verify that this pigment was betacyanin, where it can be seen that the red pigment co-migrates with beet betacyanin (Fig 4G), and that similarly treated PgDODA/CYP76AD5 co-expressing plants do not make betacyanin but only make yellow betaxanthins under these conditions (Fig 4F third from left, Fig 4G).

Phylogenetic analysis by Brockington et al. [17] indicates that CYP76AD1 is a member of a distinct clade (alpha) from CYP76AD5 and CYP76AD6 (beta). To test whether the two divergent CYP76AD1-like and CYP76AD5/6-like clades do indeed correspond to the divergent functions of CYP76AD1 and CYP76AD5/6, the activities of another member from each group was analyzed from a betalain-producing species that is not closely related to *B*. *vulgaris* (Amaranthaceae family). The sequence of *Mirabilis jalapa* (4 o’clocks; Nyctaginaceae family) MjCYP76AD3 is BvCYP76AD1-like (alpha clade) while MjCYP76AD15 is BvCYP76AD5/6-like (beta clade). Co-expression of MjCYP76AD3 and PgDODA in yeast produced red pigment (agrees with the findings of Deloache et al. [5]) and expression of MjCYP76AD15 and PgDODA produced only yellow pigment (Fig 3G), consistent with the hypothesis that the CYP76AD1-like alpha clade members are able to perform steps 1 and 3 while the CYP76AD5/6-like beta clade members just perform step 1 (Fig 1A).

## Discussion

Betalain pigments are particularly interesting from an evolutionary perspective. Questions remain about how and why they arose within a single order of flowering plants, the Caryophyllales, and why they are mutually exclusive with the almost ubiquitous anthocyanins. Given the recently identified biosynthetic steps and pathway regulatory mechanisms described here and in previous publications [1,4,5,16,30] these questions are now being addressed. Recent molecular phylogenetic analysis [17] indicates that betalains arose a single time following gene duplication and neofunctionalization of precursors of the modern *CYP76AD* and *DODA* genes.

Betalain pigments are also of interest for their many applications in the food industry, for the molecular breeding of flower and fruit colors, and, with recent discoveries, for the metabolic engineering of plant and non-plant organisms to produce these pigments. Betalains used as added or engineered food colorants may provide protection against certain oxidative stress-related disorders in humans [34–37].

Three main enzymatic steps convert tyrosine to the betalain pigment backbone. The DOPA 4,5-dioxygenase responsible for producing betalamic acid from L-DOPA (step 2; Christinet et al., 2004) and the CYP76AD1 enzyme responsible for conversion of L-DOPA to cyclo-DOPA in beet (step 3) [4] have been identified. Tyrosine hydroxylation (step 1) is the last of the three main betalain ring structure biosynthetic steps to be described at the molecular level. Deloache et al. [5] first showed that the CYP76AD1 enzyme was also able to perform tyrosine hydroxylation (thus performing both steps 1 and 3) which was confirmed by Polturak et al. [16] and these results are confirmed here. The present work demonstrates that other members of the alpha clade also have steps 1 and 3 activities and that three members of the beta clade only have step 1 activity (tyrosine hydroxylation). This confirms and extends Polturak et al.’s work on *CYP76AD6* [16]. It has been shown that yellow or golden beets are the result of mutations in *CYP76AD1* and that *CYP76AD1* lies at the beet *R* locus [4,18]. While transgenic expression of CYP76AD1 will complement yellow roots to red, performing step 3 (Fig 2A), expression of beta clade members, CYP76AD5, CYP76AD6, and CYP76AD15 will not. Thus beets appear to have a single enzyme that performs step 3 but they are at least threefold redundant for step 1. The functional divergence of the similar CYP450 genes presented here would allow betalain producing plants to selectively produce yellow or red pigment by differential regulation of the CYP450 that produces cyclo-DOPA. It has been shown that mutating the phenylalanine at position 309 to leucine in CYP76AD1 ablates its ability to produce cyclo-DOPA but leaves its ability to hydroxylate tyrosine intact [5]. It is noted here that the vast majority of beta clade members have leucine at this position with at least one member having methionine, a conservative change from leucine. It is also noted that the vast majority of the gamma clade members have leucine at this position, which may indicate that they are also tyrosine hydroxylases.

If CYP76AD1, CYP76AD5 and CYP76AD6 are the only enzymes responsible for tyrosine hydroxylation in beet, then knocking them all out should prevent any betalain pigment production. It has been shown that VIGS against CYP76AD1 and CYP76AD6 leads to loss of betalain pigmentation [16]. Based on the high sequence identity between CYP76AD5 and CYP76AD6, it’s quite possible that they were both silenced in those experiments. This triple redundancy for step one function, and possibly more redundancy, would explain the lack of a step 1 mutant in beet, and may explain that same absence in any known species. Within beet, CYP76AD5 and CYP76AD6 are the most similar paralogs to each other (~85% identity) and to CYP76AD1 (~70% identity), but there are several other paralogs in the beet genome, from the CYP76AD gamma clade, in the 50–60% amino acid identity range and all of these have leucine at the critical F309 position. This may indicate that the gamma clade members can also redundantly perform tyrosine hydroxylation, but they have not been tested. The fact that Arabidopsis expressing DODA do not produce betaxanthins indicates that there is no native tyrosine hydroxylating activity in *A*. *thaliana* [32]. However, in support of the existence of non-PPO/non-CYP76AD plant enzymes able to perform the L-DOPA to cyclo-DOPA step is the fact that *Antirrhinum majus* (snapdragon) cells expressing the *PgDODA* gene and fed L-DOPA produce not only yellow betaxanthin but also the red betacyanin pigments [32]. The arguments are that if a PPO were producing cyclo-DOPA, the same enzyme would produce LDOPA from tyrosine leading to betalamic acid, and, CYP76AD subfamily enzymes are not found outside the Caryophyllales order [17] so snapdragon would not have these enzymes. This is in contrast to beet where mutations in CYP76AD1 alone appear to knock out the ability to produce cyclo-DOPA (step 3, Fig 1) or betanin; i.e. there is no enzyme in beets other than CYP76AD1 that performs this function.

Recent phylogenetic analyses indicate that both the CYP76AD and DODA alpha and beta groups have arisen via gene duplication and subsequent diversification and that these events may underlie the origin of betalains [17]. At this point the minimum number of changes required for the functional divergence of CYP76AD1-like and CYP76AD5/6-like enzymes is not known, but polymorphisms identified from protein alignments of presumed orthologs and the mutagenesis work of DeLoache et al. [5] point to where these mutations probably lie.

It will be interesting to determine whether the CYP76AD1 (step 1 and 3 performing enzymes), or CYP76AD5/6 (step 1 only enzymes) CYP450s better represent the ancestral type. If the CYP76AD5/6 type predates the CYP76AD1 type, it would indicate that those first betalain-producing plants only had yellow betalains, which would be unlikely to supplant anthocyanins through analogous biological function. The latter could not have occurred until the new, step 3 activity evolved, providing betacyanins with hues similar to anthocyanins. If the CYP76AD1 type evolved first, this would indicate that red betalains predominated, allowing anthocyanins to be functionally replaced in many or all contexts. It would also mean that evolving CYP450s that limit the production of red while leaving yellow intact was an important subsequent evolutionary event providing a selective advantage. Of the polymorphisms that differentiate the CYP76AD1 and CYP76AD5/6 groups, the next closest orthologs (gamma group) are more similar to the CYP76AD5/6 genes, suggesting that tyrosine hydroxylation (step 1) was the ancestral function and cyclo-DOPA formation (step 3) evolved later in the CYP76AD1 lineage. Further, ancestral sequence reconstructions indicate that the ancestral copy of the alpha and beta groups would have had a leucine at the pivotal amino acid position 309, which likely would have limited its activity to step 1, thus producing only yellow pigments. This is confounded by the finding here that CYP76AD5/6 appear to have weak step 3 activity when overexpressed in yeast; however, in the beet *r* mutant, AD5/6 cannot replace CYP76AD1’s step 3 activity.

The current taxonomic distribution of betalains has been a perplexing feature of angiosperm evolution. The most recent phylogenetic analyses suggest that betalains have had a single origin in the Caryophyllales, with subsequent betalain losses and reversions to anthocyanin production. What remains unclear is why betalains may have arisen in the first place and how they supplanted the well-entrenched and successful anthocyanins. As an alternative to outright anthocyanin replacement by betalains, the betalain pathway may have evolved in a lineage that lost the ability to synthesize anthocyanins. Indeed, some extant species in the Caryophyllales reportedly make neither betalains nor anthocyanins [38]. In this scenario, betalains would not have replaced anthocyanins outright but instead evolved to compensate for the loss of red/violet pigments. Such a scenario would likely require long-term persistence of a lineage that made no or little anthocyanins. This scenario is also consistent with the absence of species that possess both pigment pathways but would also require some groups to regain the anthocyanin pathway, such as the Molluginaceae and Caryophyllaceae. Signatures of such pathway recoveries might be expected to be evident in the sequences of the anthocyanin synthesis or regulatory networks.

## Supporting Information

S1 Fig Phylogenetic analysis of the CYP76AD subfamily members used to generate the LOGO shown in Fig 1B. The tree presented here is very similar to that presented by Brockington et al. [16], except that the gamma group is polyphyletic; this may be a product of sampling as only complete or near complete coding sequences were used here. The previously defined alpha and beta groups are recovered here with strong support and the activities of those two groups are indicated by color-coded branches; the alpha group is shown in red to indicate their step 1 and step 3 roles leading to betacyanin production and the beta clade is shown in yellow to reflect their step 1 only activity, leading to yellow betaxanthins. The genes that were functionally characterized in this paper are highlighted in color as well. Node labels A, B, and C indicate where ancestral sequences were reconstructed. Each node is also labeled with calculated probability.
(TIF)

S2 Fig Amino acid alignment of the CYP76AD subfamily members used to generate the phylogenetic tree and LOGO. Amino acids that appear to differentiate the three primary groups resolved in the gene tree are highlighted. The top (alpha) group consists of the CYP76AD1-like homologs (functional steps 1 and 3); center (beta) group, CYP76AD5/6-like (functional step 1 only); bottom (gamma) group, unknown function. Red: conserved among CYP76AD1-like. Yellow: conserved among CYP76AD5/6-like. Blue: conserved among CYP76AD1, 5, and 6. Green: conserved among the unknown CYP76AD members. The numbers below the alignment are the amino acid positions in CYP76AD1 and correspond to the amino acids used in the LOGO analysis.
(DOC)

S1 Table Sequences discussed in the text and used to produce the LOGO analysis, sequence alignment, and phylogenetic tree. Shown are the species name, family, gene name, and accession number for all genes referenced in the manuscript.
(DOCX)
