# Supplementary figures and images for: Tyrosine Hydroxylation in Betalain Pigment Biosynthesis Is Performed by Cytochrome P450 Enzymes in Beets (Beta vulgaris)
Source: PLoS One. 2016 Feb 18;11(2):e0149417. doi: 10.1371/journal.pone.0149417 (PMC4758722; doi:10.1371/journal.pone.0149417)

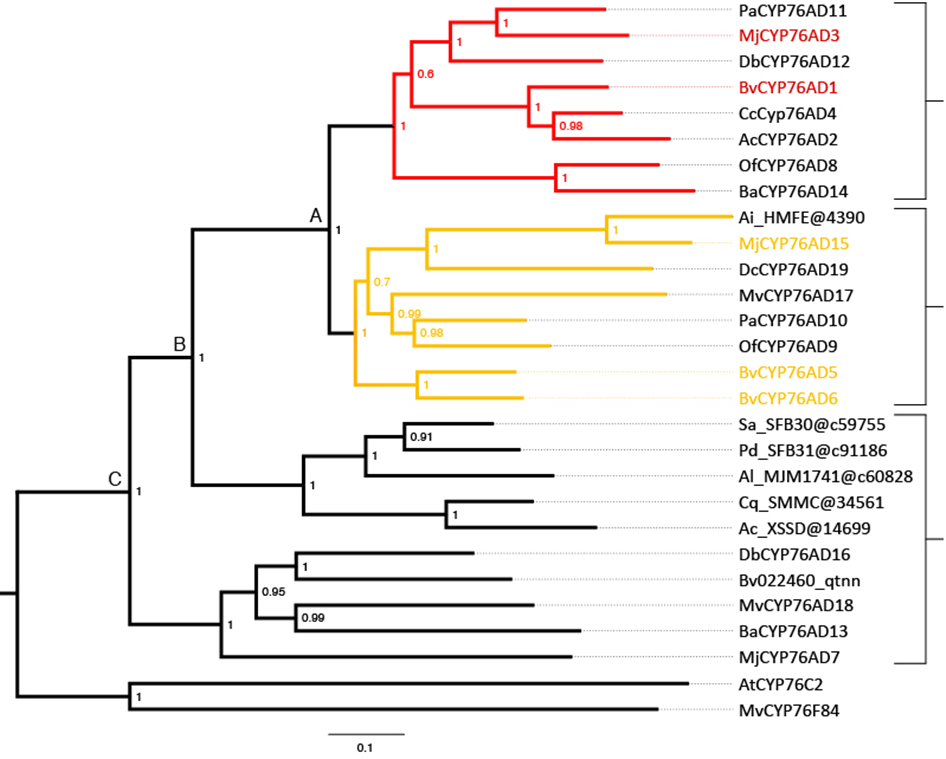

Supplement: S1 Fig — The tree presented here is very similar to that presented by Brockington et al. [16], except that the gamma group is polyphyletic; this may be a product of sampling as only complete or near complete coding sequences were used here. The previously defined alpha and beta groups are recovered here with strong support and the activities of those two groups are indicated by color-coded branches; the alpha group is shown in red to indicate their step 1 and step 3 roles leading to betacyanin production and the beta clade is shown in yellow to reflect their step 1 only activity, leading to yellow betaxanthins. The genes that were functionally characterized in this paper are highlighted in color as well. Node labels A, B, and C indicate where ancestral sequences were reconstructed. Each node is also labeled with calculated probability. (TIF) [file pone.0149417.s001.tif]
