# Supplementary material for: Tyrosine Hydroxylation in Betalain Pigment Biosynthesis Is Performed by Cytochrome P450 Enzymes in Beets (Beta vulgaris)
Source: PLoS One. 2016 Feb 18;11(2):e0149417. doi: 10.1371/journal.pone.0149417 (PMC4758722; doi:10.1371/journal.pone.0149417)
Supplement: S2 Fig — Amino acids that appear to differentiate the three primary groups resolved in the gene tree are highlighted. The top (alpha) group consists of the CYP76AD1-like homologs (functional steps 1 and 3); center (beta) group, CYP76AD5/6-like (functional step 1 only); bottom (gamma) group, unknown function. Red: conserved among CYP76AD1-like. Yellow: conserved among CYP76AD5/6-like. Blue: conserved among CYP76AD1, 5, and 6. Green: conserved among the unknown CYP76AD members. The numbers below the alignment are the amino acid positions in CYP76AD1 and correspond to the amino acids used in the LOGO analysis. (DOC) [file pone.0149417.s002.doc]

BvCYP76AD1 ---------------MDHA-TLAMI-LAIWFIS----------FHFIKLLF----SQQTT

OfCYP76AD8 ---------------MDTP-TLSYF-ISAITFYYIAFQIVKLGFNVIMTSK----KTKRR

BaCYP76AD14 ---------------MDNT-TLAIL-LS-TSYFIIYLIITKLGFHYKVLP-----KNPKQ

AcCYP76AD2 ---------------MDNA-TLAMI-LTIWFIS----------INFIKMFF----YHQNT

CcCYP76AD4 ---------------MDNA-TLAML-LAIWFIS----------FHFIKMLF----TNQST

MjCYP76AD3 ---------------MDFL-TLVMI-LSIIFFF----------YNLLKMKF----TTHSD

PaCYP76AD11 ---------------MDHT-TLAMI-LSVIFLL----------YNLVKAIF----SQ-SN

DbCYP76AD12 ---------------MDYT-TLVMI-LSIVFFC----------YNLFNLLF----TR-KN

MvCYP76AD17 ---------------MDQF-TLGMI-LSFLFVW----------FYLVKTIF----GPFGN

MjCYP76AD15 ---------------MENT-MLGVI-LATIFLT----------FHIMKMLF----SP--S

Ai_HMFE@4390 --------------------------------------------------------S--N

DcCYP76AD19 -------------------------------------------------MF----FHHPK

PaCYP76AD10 ---------------MDHT-TIAML-VSIVFLC----------FHLIKSFS----SHSKN

OfCYP76AD9 ---------------MDHA-ILATL-VCILFVC----------FHLLWR------RSYKN

BvCYP76AD5 ---------------MDNT-TLALI-LSSLFVC----------FQLIRSFI----NHAKK

BvCYP76AD6 ---------------MDNA-TLAVI-LSILFVF----------YHIFKSFF----TNS-S

MjCYP76AD7 ---------------MNHVLTIPVIFL---LII----------LIIIH------KLIFSS

MvCYP76AD18 ---------------MEYHT-FPLILIPLISTI----------IYLLK------ISTKPS

BaCYP76AD13 ---------MGNQTTMELYTTLTLFI--SIVSF----------LFLHH------FLKNSP

Bv022460_qtnn ---------------MEYFT-TLLLLLS--III----------LTILL------STKLFT

DbCYP76AD16 MLSFNNIQELCNNYQMENYTITSIVLFPIIFIL----------LPLLY------LSLKSR

Cq_SMMC@34561 ---------------------LLLL-FSFVWAF----------LYLFK-HFNSSKLTSNN

Ac_XSSD@14699 --------------------IILIL-IPFIWVW----------KL----------KSSSK

Al_MJM1741@c60828 ---------------MDGSILLALI-VSSIWVI----------FFLLKPKLSKHA---SV

Pd_SFB31@c91186 ---------------MDYS-MLAAL-LSLLWAC----------FYFLKIKSNSSKLTSIN

Sa_SFB30@c59755 ---------------MDYS-MLAAL-VSFLCAC----------FYFLKLNLSSSKLTS--

BvCYP76AD1 KL-LPPGPKPLPIIGNILEVGKKPHRSFANLAKIHGPLISLRLGSVTTIVVSSADVAKEM

OfCYP76AD8 RLPLPPGPKPLPIIGNVFELGPKPHRSFASLAKVYGPLMSLRLGSVTTIIVSSSDVAKEM

BaCYP76AD14 RLRLPPGPKPLPIIGNVLELGSKPHRSFTNLAKVHGPLISLRLGSVTTIIVSSSHVAKEM

AcCYP76AD2 KLSLPPGPKPLPIIGNILEVGKKPHRSFANLAKIHGPLISLRLGSVTTIVVSSAEVAKEM

CcCYP76AD4 KL-LPPGPKPLPIIGNILEVGKKPHRSFANLAKIHGPLISLKLGSVTTIVVSSAEVAKEM

MjCYP76AD3 -AQLPPGPKPMPIFGNIFELGEKPHRSFANLAKTHGPLMSLRLGSVTTIVVSSAEVAKEM

PaCYP76AD11 -TKLPPGPKPVPIFGNIFELGDKPHRSFANLAKIHGPLITLKLGSVTTIVVSSAEVAKEM

DbCYP76AD12 -TKLPPGPKTIPIFGNIFELGKKPHQSFANLAKIHGPLMSLKLGSVTTIVVSSAEVAREM

MvCYP76AD17 ASKIPPGPTPLPIVGNIFQLGKLPHRSFSNLSKIYGSMMTLKLGMVTTIVVSSAEVAKEM

MjCYP76AD15 KVKLPPGPRPLPIIGNILELGDKPHRSFANLAKIHGPLVTLKLGSVTTIVVSSSEVAKEM

Ai_HMFE@4390 KARLPPGPKPLPIIGNILELGEKPHRAFANLAKIHGPLITLKLGSVTTIVVSSSEVAKEM

DcCYP76AD19 NNKLPPGPKQIPIFGNIFDLGEKPHRSFTNLAKIYGPLISLKLGSVTTIVVSSSDVAREM

PaCYP76AD10 PSQLPPGPKPLPIFGNIFELGEKPHRSFARLAKIHGPLISLKLGSVTTIVVSSSEVAKEM

OfCYP76AD9 SSKLPPGPKPVPIFGNIFELGEKPHRSFADLAKIHGPLISLKLGSVTTIVVSSSDVAKEM

BvCYP76AD5 SNKLPPGPKRMPIFGNIFDLGEKPHRSFANLAKIHGPLVSLQLGSVTTVVVSSADVAKEM

BvCYP76AD6 SRRLPPGPKPVPIFGNIFDLGEKPHRSFANLSKIHGPLISLKLGSVTTIVVSSASVAEEM

MjCYP76AD7 NHKLPPGPKPWPIIGNIHLLGNKPHRSLSKLAKKYGPLISLKLGTITTIVISSPDIAKEM

MvCYP76AD18 KSNLPPGPKPWPIIGNIHLLGHKPHRSVSELSKKYGPIMTLKLGSVTTIVISSPKVAEEM

BaCYP76AD13 KSKLPPGPKPWPIIGNIHLLGSKPHCSVANLSKIYGPVMSLKLGSIVTIVISSPKVAKEM

Bv022460_qtnn KSNLPPGPKPWPIIGNILELGKLPHQAVDKLSKTYGPILSLKLGSITTIVISSPEIVKEM

DbCYP76AD16 QSKLPPGPKPWPIIGNILLLGDKPHQSVAKLSKIYGPLMSLKLGSITTIVISSPTIAKEM

Cq_SMMC@34561 NSRLPPGPKPVPILGNLPHLGDSPHRYLANLAKTYGPLISVKFGSITTIVVSSSTVAKEM

Ac_XSSD@14699 LSRLPPGPKPVPILGNLGHLGDKPHRYLASLVKTYGPLIHLKFGSITTIVVSSSAVAKEM

Al_MJM1741@c60828 YSRLPPGPKSLPIIGNLHQLGERPHRSLAELARTYGPLMYLKFGTINTIVVSSSKVAKEM

Pd_SFB31@c91186 GSRLPPGPRPMPIIGNLHQLGDTPHRTLAELSNKYGPLISLRFGSITTIVVSSSLVAKEL

Sa_SFB30@c59755 NGRLPPGPKPLPVIGNLHQLGETPHRSLAELARTYGPLICLKFGSITTIVVSSSVVAKEM

BvCYP76AD1 FLKKDHPLS-NRTIPNSVTAGDHHKLTMSWLPVSPKWRNFRKITAVHLLSPQRLDACQTF

OfCYP76AD8 FLKNDQPLSSTRTIPNSVTAGDHHKLTMSWLPVSPKWRSFRKITTFHLLSPQRLDACSGL

BaCYP76AD14 FLKNDQSLSSNRTIPHSVTAGDHHKLTMSWLPVSPKWRSFRKITTFHLLSPQRLDACCSL

AcCYP76AD2 FLKKDQPLS-NRNVPNSVTAGDHHKLTMSWLPVSPKWRNFRKITAVHLLSPLRLDACQSL

CcCYP76AD4 FLKKDQPLS-NRTVPNSVTAGDHHKLTMSWLPVSPKWRNFRKITAVHLLSPLRLDACQSL

MjCYP76AD3 FLKNDQSLA-DRSVPNSVTAGDHHKLTMSWLPVSPKWKNFRKITAVHLLSPQRLDACHAL

PaCYP76AD11 FLTNDQLLA-NRNVPNSVTAGDHHKLTMSWLPVSPKWKTFRKITAVHLLSPQRLDACQAL

DbCYP76AD12 FLKNDQLLS-NRTVPNSVTAGDHHKTTMSWLPVSQKWRNFRKITAVHLLSPQRLDSCQAL

MvCYP76AD17 FLQHDLAFS-NRMIPDSVTGGKHDKLSVTWIPVSPKWRHLRKIFALNLLSNQKLDETQSQ

MjCYP76AD15 FLKNDQPLA-NRTIPDSVRAGNHDKLSMSWLPVSPKWRNLRKISAVQLLSTQRLDASQAH

Ai_HMFE@4390 FLKNDQALA-NRTI-----QGNHDKLSMSWLPVSPKWRNLRKISAVQLLSSQRLDSSQSH

DcCYP76AD19 FLKSDHVLS-NRTIPDSVKAGNHDKLSMSWLPVSQKWRHLRKIAATQLLSTQRLDSSESL

PaCYP76AD10 FLKHDQVLA-NRTIPDSVRAGNHDKLSMSWLPVSVKWRSLRKIVVVQLFSTQRLDVSQSL

OfCYP76AD9 FLKHDQVFA-NRTIPDSVRAGNHDKLSMSWLPVSAKWRNMRKISAVQLLSTQRLDSNQGL

BvCYP76AD5 FLKNDQALA-NRTIPDSVRAGDHDKLSMSWLPVSAKWRNLRKISAVQLLSTQRLDASQAH

BvCYP76AD6 FLKNDQALA-NRTIPDSVRAGDHDKLSMSWLPVSQKWRNMRKISAVQLLSNQKLDASQPL

MjCYP76AD7 FTKHDLALS-ARQVPEAAKIADHHLYSIIFLPICPKWRGYRKISTVHLFTNQILDTSQHL

MvCYP76AD18 FLKHDLALS-GRKTPYAICVQDHDKYSMAFIPVSPKWRNLRKIATVQLLTNHRLDASQGL

BaCYP76AD13 FLKHDSACS-SRRVPDCFKIIGHEQVSMVWLPANHRWRNLRKISATQLFTNLRLDANQGL

Bv022460_qtnn FLEHDLALS-SRPSPDASRVGNHNKFSIVWLPVSPKWRDLRKIATIQLFTTQRLDSSQEL

DbCYP76AD16 FLKHDLAFS-SRNVPDVRTIDNHDKLSIVWLPVCPKWRDLRKIATIQLFTNQRLDATRDL

Cq_SMMC@34561 FQKHDLTLS-TRHVSAAVRSNGHDKFSVAWLPVCAKWRTLRKISALQLFSTQRLDASQAL

Ac_XSSD@14699 FQKHDLTVS-SRQVCAALRANGHDQFSVVWLPVCAKWRSLRKISAIYLFSSQRLDASQAL

Al_MJM1741@c60828 FQKHDLSLA-NRKVPAAVRANGHDMYSIAWLPICPKWRTLRKISAVHSLSNQRLDASQSF

Pd_SFB31@c91186 FQKHDLALA-NRKVPAAVRSNEHDKYSIAWLPVCPKWRNLRKISAVHSLSTQRLDASQSL

Sa_SFB30@c59755 FQKHDLSLA-NRKVPAAVRANDHDKFSIAWLPVCPKWRNLRKISAIHSLSNQRLDDSQSL

103

106

111

114

122

127

131

134

138

144

BvCYP76AD1 RHAKVQQLYEYVQECAQKGQAVDIGKAAFTTSLNLLSKLFFSVELAHH-KSHTSQEFKEL

OfCYP76AD8 RQAKVQQLYEYVLECSRTGQAVDIGKAAFTTSLNLLSKLFFSLELANH-TSDKSQEFKEL

BaCYP76AD14 RQAKVQQLFEYVLQCSRTGQPVDIGKAAFTTSLNLLSKLFFSLELAHH-RSTKSQEFKDL

AcCYP76AD2 RHAKVQQLYQYVQECALKGQSVDIGKAAFTTSLNLLSKLFFSKELACH-KSHESQELKQL

CcCYP76AD4 RHAKVQQLFQYVQECAQKGQAVDIGKAAFTTSLNLLSKLFFSKELASH-KSRESQEFKQL

MjCYP76AD3 RHAKVKQLYEYVQECALKGEAVDIGKAAFTTSLNLLSNLFFSVELANH-TSNTSQEFKQL

PaCYP76AD11 RHTKVKQLYEYVQECAKRGEAVDIGKAAFTTSLNLLSNLFFSVELANH-TSSSSQEFKEL

DbCYP76AD12 RQAKVKQLFNYIHECAQKGEAVDIGKAAFTTSLNLLSNLFFSVELANH-KSSSSQEFKQL

MvCYP76AD17 RQAKVQQLLEYVQECAKKGCAVDIGRATFTTTLNLLSNTVFSMELAHY-SSNASQEFKRL

MjCYP76AD15 RQAKIKQLIEYVKKCSKIGQYVDIGQVAFTTSLNLLSNTFFSKELASF-DSDNAQEFKQL

Ai_HMFE@4390 RQSKVKQLVDYVAECSKLGQSVDIGRVAFTTSLNLLSNTFFSKELASF-DSNNAQEFKNL

DcCYP76AD19 RQAKVEKLLEYVQECCNSGLSVDIGRAVFTTTLSLLSNTFFSKELANY-SSSKSQEFKQL

PaCYP76AD10 RHAKVQQLHEYITECSKKGEPVDIGRAAFTTSLNLLSNTFFSMELANH-SSSASQEFKQL

OfCYP76AD9 RQAKVQQLLEYVQECCKKGQPVDIGRAAFTTSLNLLSNTFFSMELAQH-SSSASQEFKQL

BvCYP76AD5 RQSKVQQLLEYVHDCSKKGQPVDIGRAAFTTSLNLLSNTFFSVELASH-ESSASQEFKQL

BvCYP76AD6 RQAKVKQLLSYVQVCSEKMQPVDIGRAAFTTSLNLLSNTFFSIELASH-ESSASQEFKQL

MjCYP76AD7 RRKKVHELVEYIKCCCENNQVVEIGKAAFTTSLNLLSNTFFSFDLASDIDFGYSIKFKNI

MvCYP76AD18 RREKVHELVQYARDCCEKGIAMDIGKAGFITSLNLLSNTFFSMNLTSY-DSSFSGEFRDI

BaCYP76AD13 RREKINDLIQFVKDSCGSGLAIDIGKAAFTTSLNLLSNTFFSVELASY-DSSISHEFKEL

Bv022460_qtnn RQIKVNELVDYVRQCCEKGLPVDVGKAGFTTTLNMLSNTFFSMDLASH-ASSNSQEFKDL

DbCYP76AD16 RRKKVEELVGYARQCCEQGLALDIGKAAFTTSLNLLSNTFFSMNLGSH-DSSWSQEFKEL

Cq_SMMC@34561 RQEKVSKLMEYVKECSKSGEAIDVGGVAFTTSLNLLSNTFFSFDLASY-NSSDTGEFKEL

Ac_XSSD@14699 RQEKVSKLMEYVNECCKKGEALDIGVAAFTTSLNLLSNTFFSFDLAGY-SSRDSGEFKEL

Al_MJM1741@c60828 RQAKVADLLEYVKGCSVTGEVVDIGKAAFTTSLNLLSNTFFSFDLASH-RSSDSGQFKDL

Pd_SFB31@c91186 RQAKVMELLEFVRGCCKAGEAVDVGKAAFTTSLNLLSNTFFSFDLASH-SSSDSGEFKEL

Sa_SFB30@c59755 RQAKVLELLDYVRQCCKVGEAVDVGKAAFTTSLNLLSNTFFSLDLASH-SSTDSGEFKEL

156

174

186

BvCYP76AD1 IWNIMEDIGKPNYADYFPILGCVDPSGIRRRLACSFDKLIAVFQGIICERLAPDSS--TT

OfCYP76AD8 IWNIMEDIGKPNYADYFPCLKYFDPSGIRRRLACSFEKLIEVFQVIIRQRLSLSSS---G

BaCYP76AD14 IWNIMEDIGKPNIADHFPCLKYFDPSGIRRRLASSFERLIEVFQDIIRQRMSLSFG----

AcCYP76AD2 IWNIMEDIGKPNYADYFPILGCIDPLGIRRRLAANFDKLISVFQTIISERLENDIN----

CcCYP76AD4 IWNIMEDIGKPNYADYFPILGCVDPSGIRRRLASNFDKLIEVFQCIIRQRLERNP-----

MjCYP76AD3 IWDIMEDIGKPNYADYFPLLKYVDPSGIRRRLAANFDKLIDVFQSFISKRLSSAY----S

PaCYP76AD11 IWDIMEDIGKPNYADYFPVLKCVDPWGIRRRLESNFDKLIEVFQSFIRKRLSTEPF--SA

DbCYP76AD12 IWNIMEDIGKPNYADYFPVLKYVDPSGIRRRLASNFNKLIDVFQGFIRLRMSTNS---SC

MvCYP76AD17 MWCIMEEIGRPNYADYFPFLRHFDPFGNRRRLTAAYAKLIGFFENIVHERLNARSAKL--

MjCYP76AD15 MWCIMEEIGRPNYADYFPILGYVDPFGARRRLSRYFDQLIEVFQVIIRERLTHDNNIV--

Ai_HMFE@4390 MWCIMEEIGRPNYADYFPILGYVDPFGVRRRLSRYFDQLIEVFQE----RLAKNSNNV--

DcCYP76AD19 MWDIMVEIGRPNYADYFPMLGFMDPFSIRRRLSSYFDKLIGVFQEIISERLSLRKK--SC

PaCYP76AD10 MWCIMEEIGRPNYADFFPILGYVDPFGIRRRLAVYFDKLIAVFQEIIRERQKARFTNL--

OfCYP76AD9 MWCIMEEIGRPNYADYFPILGYFDPFGIRRRLTAYFDKLIAIFQDIIHERLKARSTG---

BvCYP76AD5 MWNIMEEIGRPNYADFFPILGYLDPFGIRRRLAGYFDQLIAVFQDIIGERQKIRSANLSG

BvCYP76AD6 MWNIMEEIGRPNYADFFPILGYIDPFGIRRRLAGYFDKLIDVFQDIIRERQKLRSSNSSG

MjCYP76AD7 VGELSEALAKPNLSDFFPIIKSLDLQGVKKKMEVLFTDMWDVFRKVVEERLSDNSK----

MvCYP76AD18 VWNMLEEAGRPNLSDFFPVLKGLDLQGMRKRYSVYYYKMIAIFDEIINQRLKDS-T----

BaCYP76AD13 VWRVLEVGATPNISDFFPLIRTFDLQGTRRRGKYYLLKLRGIFEKIIDERLSCQ-T----

Bv022460_qtnn VWSLLEEGAKPNVSDFFPIVRELDLQGVSKNRRVHMKKLMGIFEEIIDGRLTKL-K----

DbCYP76AD16 VWNLLEEGAKPNVSDYFPILKRFDLQGAVRRVSRYVDKLMAVFEDIIDERLKNS-T----

Cq_SMMC@34561 VWKIMEEIGKPNLVDCFPMLRFLSVFSVNCNLMGYGNKLNEVFVDIINKRLESFGV---S

Ac_XSSD@14699 VWKIMEEIGKPNWVDCFPMLRFFSKFSVNRKLMGYGNKLNEIFEDIIQKRLNDCDV---C

Al_MJM1741@c60828 VWKIMEEIGKPNLADCFPMLRFFSSISVNQQLMGYGNKLNEVFADIINERLKNGVSN---

Pd_SFB31@c91186 VWKIMEEIGKPNLADCFPVLRFVSAVSVNCQLMGYGNKLNEVFAGIIEERLRSGASSD-S

Sa_SFB30@c59755 VWRIMEEIGKPNLADCFPVLRFISAVSVNSQLMGHSNKLNKVFAEIINERLKTPS--S-A

207

213

216

232

BvCYP76AD1 TTTTTDDVLDVLLQLFKQN--ELTMGEINHLLVDIFDAGTDTTSSTFEWVMTELIRNPEM

OfCYP76AD8 TNDHNNDVLDVLLDLYQQK--ELSMEEINHLLVDIFDAGTDTTSSTFEWAMAELIKNPRM

BaCYP76AD14 -SSHNNDVLDVLLGLYNQK--ELTMDEINHLLVDIFDAGTDTTSSTFEWSMAELMKNRRI

AcCYP76AD2 SNATTNDVLDVLLQLYKQK--ELSMGEINHLLVDIFDAGTDTTSSTFEWVMAELIRNPKM

CcCYP76AD4 STPPTNDVLDVLLELYKQN--ELSMGEINHLLVDIFDAGTDTTSSTFEWVMAELIRNPEM

MjCYP76AD3 SATSLDDVLDVLLKLLKEK--ELNMGEINHLLVDIFDAGTDTTSNTFEWAMAELMRNPIM

PaCYP76AD11 SAKTPNDVLDVLLNLLKEE--ELNMGEINHLLVDIFDAGTDTTSSTFEWAMAELVRNPEM

DbCYP76AD12 GATNPNDVLDVLLNLYKGD--DLNMDEINHLLVDIFDAGTDTTSSTFEWAMAELVKNPKM

MvCYP76AD17 --STTNDVLDTLLNLHQDN--QLTMDEIYHILVDIFDAGTDTTANTLEFAMAQLVKNPET

MjCYP76AD15 --GNNNDVLATLLDLYKQN--ELTMDEINHLLVDIFDAGTDTTASTLEWAMSELIKNPHI

Ai_HMFE@4390 --DIKSDILATLLDLYKQN--ELSMDEINHLLVDIFDAGTDTTASTLEWAMAELIKNPHI

DcCYP76AD19 GKDNTNDVLSTLLNLYEEN--ELSMDEVNHLLVDIFNAGTDTIASTLEWAMSELLKNPNI

PaCYP76AD10 --STTNDVLDTLLNLYQEN--ELSMDEINHLLVDIFDAGTDTTASTMEWAMAELVKNPDI

OfCYP76AD9 -SSSTNDILDTLLNLYQEN--ELSMDEINHLLVDIFDAGTDTTASTLEWAMAELVKNPEI

BvCYP76AD5 GKQTTNDILDTLLNLYDEK--ELSMGEINHLLVDIFDAGTDTTASTLEWAMAELVKNPDM

BvCYP76AD6 AK-QTNDILDTLLKLHEDN--ELSMPEINHLLVDIFDAGTDTTASTLEWAMAELVKNPEM

MjCYP76AD7 --GLKDDVLDTLLKLVDEQ--EISLDEVVHFIMDLFSAGTETTLITLEWAMTELLRCPDK

MvCYP76AD18 --SDKDDVLGSLLKLVKED--ELSLDDVRHLLLDLFIAGTDTTSATLEWAMTELLVNPQK

BaCYP76AD13 --SGKDDVLDTLLKLVKQN--ELSLHEVQHFLIDLFVAGTDTTSSVLEWAMTELLRNPEK

Bv022460_qtnn --DVKDDVLSTLLKLVKDE--ELNLDDVKHMLMDLFLAGTDTTSITLEWAMTELLRNPEK

DbCYP76AD16 --EAKDDVLNLLINLVKEN--ELSLHNVKHMLFDLFLAGTDTTSSTVEWAMAELQRHPEK

Cq_SMMC@34561 GNNDGGDVLDTLIKIMKENESELSLDDIKHLLMDFFTAGTDTTSSTLEWAMTELLHSPEK

Ac_XSSD@14699 E--NYGDVLDTLLRLMKEKESGLTLADIKHLLMDFFTAGTDTTSSTLEWALTELLHNPEK

Al_MJM1741@c60828 CADNDADVLDTLLRLMK-NDSELCLDDIKHLLMDFFTAGTDTTSSTLEWAMTELLHDPEK

Pd_SFB31@c91186 SGVGDGDVLDTLLRLMKENDSELSLDDIKHLLMDFFTAGTDTTSSTLEWAMTELLRNPEK

Sa_SFB30@c59755 SAITGGDVLDTLLRLMEENDSELSFDDINHLLMDFFTAGTDTTSSTLEWAMTELLHNPEK

275

306

309

BvCYP76AD1 MEKAQEEIKQVL---GKDK--QIQESDIINLPYLQAIIKETLRLHPPTVFLLPRKADTDV

OfCYP76AD8 METAQAEIKLIL---GKDL--HIQESDIPKLPYLRAIIKETLRLHPPTVFLLPRKADADV

BaCYP76AD14 MEKAQAEILHVL---GKNS--YIQESDISKLPYLRAIIKETLRLHPPTVFLLPRKADADV

AcCYP76AD2 MEKAQQEIHEVL---GKDR--QIQESDIIKLPYLQALIKETLRLHPPTVFLLPRKADMDV

CcCYP76AD4 MAKAQDEIEQVL---GKDR--QIQESDIIKLPYLQAIIKETLRLHPPTVFLLPRKADTDV

MjCYP76AD3 MKRAQNEIALVL---GKDN-ATIQESDIANMPYLQAIIKETLRLHPPTVFLLPRKAITNV

PaCYP76AD11 MKKAQDEIEQVL---GKDA--IIQESDIPKMPYLQAIIKETLRLHPPTVFLLPRKASSNV

DbCYP76AD12 MKKAQAEIQQVL---GKDS--IIRESDIPNMPYLQAIIKETLRLHPPTVFLLPRKADADV

MvCYP76AD17 LVKAQTEIEQAL---GKSS-SIIQESDISKLPYLQAIIKETFRLHPPAVFLLPRKAETDI

MjCYP76AD15 MAKAQEEVRRATMSHGGATVAEIQESDINNLPYIQSIIKETLRLHPPTVFLLPRKADVDV

Ai_HMFE@4390 MAKAKAEVRQVTMSRGESTLAQIEESDISNLPYIQAIVKEALRLHPPTVFLLPRKADMDV

DcCYP76AD19 MTKAQIEIKNAL---ENNSS-KIQESDIPKLPYLQAIIKETLRLHPPTVFLLPRKADEDV

PaCYP76AD10 MLKARIEIKQAL---GNDSSLIIQESDIAKLPYLQAIVKETLRLHPPTVFLLPRKAEANV

OfCYP76AD9 MIKAQDEINAAL---GKDCS-VIHESDIVKLPYLQAIVKETLRLHPPTVFLLPRKADLDV

BvCYP76AD5 MVKVQDEIEQAI---GKGCS-MVQESDISKLPYLQAIIKETLRLHPPTVFLLPRKADADV

BvCYP76AD6 MTKVQIEIEQAL---GKDCL-DIQESDISKLPYLQAIIKETLRLHPPTVFLLPRKADNDV

MjCYP76AD7 MAKAQAEIDQVI---DMDG--SVQESDIPKLPYIQAIVKEILRLHPATPFLVPRMAEQEV

MvCYP76AD18 IEIAQKQISQVF---AKDQ--VIHESDIPKLPYIHAVVKETFRMHPSVPFLLPHWAENDV

BaCYP76AD13 MGKLQNEINNAF---GNDQSKSIQESDISKLPYLQGVVKETLRLHPPAPFLLPRKVEKDI

Bv022460_qtnn MEKVQIELDKVL---GKDS--SLQESMISKLPYIQAIVKETLRLHPPTPFLIPHKAEKDV

DbCYP76AD16 MLKAQAEIDQVI---PNDG--FVQEMDISKLAYIQAIVKETLRLHPPAPFLIPHKTVKDV

Cq_SMMC@34561 MAKAQAELKQVL---GENR--IVGESDISKLPYLQAIVKETLRMHPPTVFLLPRKATNDV

Ac_XSSD@14699 MANVQAELDEVI---GKNK--VVGESDISKLPYLQATVKETLRMHPPTVFLLPRKANNEV

Al_MJM1741@c60828 MIKAQAELKQVL---GNDNL-MVLESDIPSLPYLQAIVKETLRMHPPTVFLLPRQADADV

Pd_SFB31@c91186 MAKAQAELEQVL---GNDNV-AVQESDTLKLPYLEAIVKETLRMHPPTVFLLPRKANADV

Sa_SFB30@c59755 MAKAQAELEQVL---GNDNV-LVQESDISKLPYLQSIVKETLRMHPPTVFLLPRKADADV

BvCYP76AD1 ELYGYIVPKDAQILVNLWAIGRDPNAWQNADIFSPERFIG---CEIDVKGRDFGLLPFGA

OfCYP76AD8 ELYGYTVPKNAQILVNLWALGRDPKVWENPDVFSPERFLG---CDIDVKGRNFGLLPFGA

BaCYP76AD14 ELYGYVVPKDAQILVNLWALGRDPAVWENPDEFSPDRFMG---SEIDVKGRDFGLLPFGA

AcCYP76AD2 ELYGYVVPKDAQILVNLWAIGRDSQVWEKPNVFLPERFLG---SDVDVKGRDFGLLPFGA

CcCYP76AD4 ELYGYIVPKDAQILVNLWAIGRDSQAWENPKVFSPDRFLG---CEIDVKGRDFGLLPFGA

MjCYP76AD3 KLYGYIVPKNAQILVNLWAIGRDPKVWKNPNEFLPDRFLN---SDIDVKGRDFGLLPFGA

PaCYP76AD11 ELYGYVVPKNAQILVNLWAIGRDPTVWDNPNMFSPERFLN---SDIDVKGRDFGLLPFGA

DbCYP76AD12 ELYGYVVPKNAQILVNLWALGRDPLVWKSPNVFKPERFLG---SEIDFKGRDFGLLPFGA

MvCYP76AD17 TLCGYVVPKNAQIMLNLWSIGRDPKVWQNPEVFSPERFLN---CDIDVRGRHFELLPFGA

MjCYP76AD15 QLFGYVVPKNAQVLVNLWAIGRDPNVWPDPEVFSPERFMD---CEIDVKGRDFELLPFGA

Ai_HMFE@4390 QLYGYVVPKNAQVLVNLWAIGRDPNVWSNPELFRPERFMD---CEIDVKGRDFELLPFGA

DcCYP76AD19 ELFGYIVPKNAQVLVNLWAIARDEKAWENPELFNPERFLG---RNIDVKGRNFELLPFGA

PaCYP76AD10 QLYGYLIPKNAQLLVNLWAIGRDPNVWNDPLVFSPERFLASSSCEIDVKGRHFELLPFGA

OfCYP76AD9 ELYGYVVPKNAQILVNLWAIGRDPKVWSNPEVFSPERFLD---STIDVKGRDFELLPFGA

BvCYP76AD5 ELYGYVVPKNAQVLVNLWAIGRDPKVWKNPEVFSPERFLE---SNIDYKGRDFELLPFGA

BvCYP76AD6 ELYGYVVPKNAQVLVNLWAIGRDPKVWKNPEVFSPERFLD---CNIDYKGRDFELLPFGA

MjCYP76AD7 QLCDYYVPKNAQILVNVWLIGRDPSVWSNPETFIPERFLG---RDIDVKGQDFELIPFGS

MvCYP76AD18 QLSSYYVPKDAQIWVNVWSMGRDPSVWLDPNSFIPERFLD---KDIDVKGRDFELLPFGA

BaCYP76AD13 DLVGYHVPKNSTIWVNVWSMGRDPSNWSNPEVFMPERFLD---SEIDVKSRHSELIPFGA

Bv022460_qtnn LLCNYLVPKNSIIWVNLWSIARSPSVWPNPESFSPERFLE---MEIDIKGRDFKLIPFGS

DbCYP76AD16 QLCDYTVPKNALVWVNVWSIGRDPSVWTDPDSFVPERFLE---REIDFKGRNFELIPFGA

Cq_SMMC@34561 ELYGYVVPKNAQVFVNLWAISRDPNHWVNPDLFSPERFLE---REIDMKGQDFGLIPFGA

Ac_XSSD@14699 ELYGYVVPKDAQIFVNLWAISRDPNHWTDPDLFSPERFLE---REIDMKGQDFGLIPFGA

Al_MJM1741@c60828 ELYGYLVPKNAQIFVNLWAISRDPNAWENPDVFIPERFLG---RDIDMKGQDFGLIPFGS

Pd_SFB31@c91186 DLYSYLVPKNAQVLVNLWAISRDPNTWENPNVFSPERFLD---RDIDMKGQDFGLIPFGA

Sa_SFB30@c59755 ELYGYVVPKNAQVLVNLWAISRDPNAWENPDVFSPERFLD---RDIDMKGQDFGLIPFGA

428

BvCYP76AD1 GRRICPGMNLAIRMLTLMLATLLQFFNWKLEG-----DISPKDLDM---DEKFG-IALQK

OfCYP76AD8 GRRICPGMNLAYRMLTLMLATLLQSFDWKLPN-----EMNPQNLDM---DEKFG-IALQK

BaCYP76AD14 GRRICPGMNLAIRMLTLMLATLLRSFDWKLPE-----GEAPAQLDM---DEKFG-IALQK

AcCYP76AD2 GKRICPGMNLAIRMLTLMLATLLQFFNWKLED-----GMNPQDLDM---DEKFG-IALQK

CcCYP76AD4 GKRICPGMNLAIRMLTLMLATLLQFFNWKLQD-----GMSLEDLDM---EEKFG-IALQK

MjCYP76AD3 GRRICPGMNLAYRMLTLMLATLLQSFDWKLPH-----RNSPLDLDM---DEKFG-IALQK

PaCYP76AD11 GRRICPGMNLAYRMLTLMLATLLQSFDWKLGD-----GVNPKDLDM---DEKFG-IALQK

DbCYP76AD12 GRRICPGMNLAYRMLTLMLATLLQSFDWKVAD-----GTNPQDMDM---DEKFG-IALQK

MvCYP76AD17 GRRICPGITLSYRMLHLMLAALIRSFDWKLED-----GTNPKDLDV---TEKFG-IALQK

MjCYP76AD15 GRRICPGLSLAYRMLNLMLANMVHSFDWKLPGVENGSGSEMDSLDM---DEKFG-IALQK

Ai_HMFE@4390 GRRICPGLSLAYRMLNLMLANLIHSFDWKLPDI----GSDVGELDM---DEKFG-ITLQK

DcCYP76AD19 GRRICPGVTLANRMMNLMLANLIHSFDWKLEE-----GVNPSDLDM---NEKFG-ITLQK

PaCYP76AD10 GRRICPGLTLAYRMLNLMLVTLVNSFDWKLED-----VTSAKDLDM---DEKFG-ITLQK

OfCYP76AD9 GRRICPGLTLASRMLNLMLATLVHNFNWKLED-----GMIPKDLDM---TEKFG-ITLQK

BvCYP76AD5 GRRICPGLTLAYRMLNLMMANFLHSYDWKLED-----GMHPKDLDM---DEKFG-ITLQK

BvCYP76AD6 GRRICPGLTLAYRMLNLMLATLLQNYNWKLED-----GINPKDLDM---DEKFG-ITLQK

MjCYP76AD7 GRRICPGMSLGYRMVHLMLANLLHSFDWSLPN-----GLDPKDLDM---EDTFG-LTLRK

MvCYP76AD18 GRRICPGLPLAYRMVHLTLATLLHSFDWKLGN-----GMSQVWDHVAKISAAYGGPTLQM

BaCYP76AD13 GRRICPGLPLAYRMVHLMLANLVQFFNWKVDR-----WSNPEEIDM---EEKFG-ITLQK

Bv022460_qtnn GRRMCPGMPLAYRMTHMLLATLLHSFNWKYGE------ASPKDIDM---KEKFG-LTLQK

DbCYP76AD16 GRRTCPGMPLAYRMTHLMLATLLHSFDWKLSD-----GVNPENLDM---EEKFG-ITVQK

Cq_SMMC@34561 GRRICPGDTLAFRMLNLMLGNLLHAFDWKVGD-----GLIPEDLDM---TDKFG-ITIQK

Ac_XSSD@14699 GRRICPGDTLAIRMLNLMLGNLLHGFNWKVGD-----GIRPEDLDM---SDKFG-ITIQK

Al_MJM1741@c60828 GRRICPGLTLAYRMLNLMLGTLIHAFHWELAN-----GLSPKDLDM---MDKFG-ITIQK

Pd_SFB31@c91186 GRRICPGLALAHRMLNLMLGTLIHAFNWKLGD-----GLNPEDLDM---TDKFG-ITIQK

Sa_SFB30@c59755 GRRICPGLTLAYRMLNLMLGTLIHSFNWKLGD-----GLSPEDLDM---TDKFG-ITIQK

482

451

442-443

BvCYP76AD1 TKPLKLIPIPRY----

OfCYP76AD8 TKPLQIIPLSKD----

BaCYP76AD14 TTPLKIIPIFKNSI--

AcCYP76AD2 NKPLEIIPSLRH----

CcCYP76AD4 TKPLRIIPVSRY----

MjCYP76AD3 TKPLEIIPLIKY----

PaCYP76AD11 TKPLQVIPVLKY----

DbCYP76AD12 TTPLQIIPVYKY----

MvCYP76AD17 AEPLEIVPVPKTSLIA

MjCYP76AD15 TKP-------------

Ai_HMFE@4390 VKPLQIIP--------

DcCYP76AD19 VIPLKLTPIAKL----

PaCYP76AD10 VKPLQLIPIPKK----

OfCYP76AD9 VNPLQVIPIQKN----

BvCYP76AD5 VKPLQVIPVPRK----

BvCYP76AD6 VKPLQVIPVPRN----

MjCYP76AD7 AQPLDAVAHYRS----

MvCYP76AD18 IR--------------

BaCYP76AD13 AKPLEVVPILR-----

Bv022460_qtnn AQPLQAIPIPR-----

DbCYP76AD16 VQPLQLIPAVRGSRK-

Cq_SMMC@34561 AIPLRAIP--------

Ac_XSSD@14699 ALPLRAIP--------

Al_MJM1741@c60828 EKPLLAIPISK-----

Pd_SFB31@c91186 AKPLRAIPIPK-----

Sa_SFB30@c59755 AKPLRAIPVAK-----

486
