# Supplementary material for: Tyrosine Hydroxylation in Betalain Pigment Biosynthesis Is Performed by Cytochrome P450 Enzymes in Beets (Beta vulgaris)
Source: PLoS One. 2016 Feb 18;11(2):e0149417. doi: 10.1371/journal.pone.0149417 (PMC4758722; doi:10.1371/journal.pone.0149417)
Supplement: S1 Table — Shown are the species name, family, gene name, and accession number for all genes referenced in the manuscript. (DOCX) [file pone.0149417.s003.docx]

**S1 Table. Sequences discussed in the text and used to produce the LOGO analysis, sequence alignment, and phylogenetic tree.** Shown are the species name, family, gene name, and accession number for all genes referenced in the manuscript.

| **Species** | **Family** | **Gene** | **GenBank Accession Number** |
| --- | --- | --- | --- |
| *Beta vulgaris* | Amaranthaceae | PPO | KR337592 |
|  |  | ACTIN | HQ656028 |
|  |  | CYP76AD1 | HQ656024 |
|  |  | CYP76AD5 | KM592961 |
|  |  | CYP76AD6 | KM592962 |
|  |  | Bv022460_qtnn | KQ090199.1 |
| *Amaranthus cruentus* | Amaranthaceae | CYP76AD2 | HQ656025 |
|  |  | Ac_XSSD@14699 | KR376441.1 |
| *Celosia cristata* | Amaranthaceae | CYP76AD4 | KC857455 |
| *Chenopodium quinoa* | Amaranthaceae | Cq_SMMC@34561 | KR376438.1 |
| *Mirabilis jalapa* | Nyctaginaceae | CYP76AD3 | HQ656026 |
|  |  | CYP76AD7 | KM516790 |
|  |  | CYP76AD15 | KM516798 |
| *Opuntia ficus-indica* | Cactaceae | CYP76AD8 | KM516791 |
|  |  | CYP76AD9 | KM516792 |
| *Phytolacca americana* | Phytolaccaceae | CYP76AD10 | KM516793 |
|  |  | CYP76AD11 | KM516794 |
| *Phytolacca dioica* | Phytolaccaceae | Pd_SFB31@c91186 | KR376427.1 |
| *Seguieria aculeata* | Phytolaccaceae | Sa_SFB30@c59755 | KR376422.1 |
| *Dorotheanthus bellidiformis* | Aizoaceae | CYP76AD12 | KM516795 |
|  |  | CYP76AD16 | KM516799 |
| *Basella alba* | Basellaceae | CYP76AD13 | KM516796 |
|  |  | CYP76AD14 | KM516797 |
| *Mollugo verticillata* | Molluginaceae | CYP76AD17 | KR779855 |
|  |  | CYP76AD18 | KR779856 |
|  |  | CYP76F84 | KR779857 |
| *Dianthus caryophyllus* | Caryophyllaceae | CYP76AD19 | KR779858 |
| *Arabidopsis thaliana* | Brassicaceae | CYP76C2 | NM_130119.3 |
